# Experimental and Simulation Study of the Effect of Plastic Residual Strain on the Electrochemical Corrosion of Biomagnesium Alloys

**DOI:** 10.3390/ma18112482

**Published:** 2025-05-25

**Authors:** Xinqi He, Chao Xie

**Affiliations:** Faculty of Mechanical Engineering and Mechanics, Ningbo University, Ningbo 315211, China; hexinqi0127@126.com

**Keywords:** ZK60 magnesium alloy, plastic residual strain, phase field, electrochemical corrosion, interface kinetic coefficient

## Abstract

In this study, the effect of plastic residual strain on the corrosion behavior of ZK60 magnesium alloy was systematically revealed using a research method combining experimental characterization and numerical simulation. Based on the multiphysical field coupling theory, a numerical model containing deformation field, corrosion phase field, and material transfer field was constructed, and the dynamic simulation of plastic residual strain-induced corrosion damage was successfully realized. Tafel polarization curves obtained from electrochemical tests were fitted to the key parameters of the secondary current distribution. The kinetic parameter *L* controlling the corrosion rate in the phase-field model was innovatively determined by the inverse calibration method, and a quantitative relationship between the kinetics of electrochemical corrosion and the phase-field theory was established. The corrosion depth distribution of the pre-strained specimens is quantitatively characterized and the results are in agreement with the finite element simulation results. The coupled strain-corrosion analysis method proposed in this study provides a theoretical basis for the design and life prediction of corrosion resistance of components under complex stress states.

## 1. Introduction

Bone tissue defect repair, a major challenge in the global medical field, has now become the second largest demand for human tissue transplantation. Although autograft and allograft are still the mainstream clinical techniques, they are limited by the shortage of bone sources and the risk of immune rejection, respectively, making bone tissue engineering the most promising alternative. While traditional bone tissue engineering is limited by cellular interference effects, biomimetic multilayered bone scaffolds have significantly improved bone regeneration efficacy through the construction of a three-dimensional biomimetic microenvironment, tunability of mechanical properties, and multicellular interaction regulation. However, the optimization of scaffold materials is still the key bottleneck, and novel material systems with mechanical suitability, controlled degradation, and bioactivity need to be developed.

Magnesium alloys are ideal candidates for orthopedic implants due to their light weight, high specific strength and specific stiffness, and elastic modulus matched to bone tissue [1,2,3]. Its biodegradable properties can avoid secondary surgery [4], and the released magnesium ions can promote bone cell proliferation and angiogenesis. However, the high electrochemical activity of magnesium leads to its excessive corrosion rate in Cl^−^ containing physiological environments, and the risk of uncontrolled degradation is further exacerbated by the loose oxide film on the surface. In particular, the electrochemical activity of magnesium increases under high plastic residual strain, and at the same time, the strain will lead to an increase in dislocation density, lattice distortion, etc. These structural defects for the penetration of corrosive media provides a channel, accelerating the corrosion process.

In recent years, researchers have tackled this challenge in a multidimensional way through material modification methods, mainly including alloying modification and surface treatment. Alloying modification is to alloy biocompatible metals by adding elements such as Zn, Mn, and Zr to improve the corrosion resistance [5,6,7,8,9]. While surface treatment is mainly to develop surface protection methods such as micro-arc oxidation, chemical conversion film, and polymer coating [10,11,12,13], the synergistic innovation of the two methods has significantly enhanced the material properties. Nonetheless, its plastic deformation capacity is poor, and it is prone to introduce plastic residual strain in conventional manufacturing and processing, that is, the irrecoverable permanent deformation that remains when the material is unloaded after undergoing plastic deformation. This strain can significantly increase the localized corrosion susceptibility of the material, which in turn induces localized corrosion. However, the coupling mechanism between plastic residual strain and corrosion behavior remains to be investigated, and the mechanism of non-uniform pre-strain in physiological environments on the mechanical properties of alloys is difficult to describe quantitatively.

The degradation of mechanical properties of magnesium alloys in corrosive environments has been a difficult research problem [14,15]. Most of the existing models are calculated using the quadratic current method, however, it is difficult to avoid the need to use a dynamic mesh to calibrate the movement of the corrosion interface during the calculation process of finite element software, which leads to inherent defects such as computational inefficiency of the model and poor adaptability to three-dimensional complex structures. The phase-field method, as an emerging numerical simulation method, does not require the use of dynamic mesh, which is a good solution to the above problems. By describing the corrosion process as a continuous evolution of phase-field variables, the phase-field method can effectively simulate the local damage behavior of magnesium alloys in corrosive environments, providing a new tool for understanding their corrosion mechanisms. In recent years, the expansion of the phase-field method in the field of ductile fracture and its combination with elastoplastic and thermoplastic mechanics have further enhanced its applicability in the simulation of complex material behavior [16,17]. Researchers have successfully captured the localized fracture behavior of magnesium alloys in corrosive environments by defining the fracture toughness as a function of accumulated plastic strain [18]. This type of model not only considers the role of elastic strain potential energy, but also introduces the effect of plastic strain on the fracture process, which can more accurately describe the damage evolution process of magnesium alloys under the combined effect of corrosion and mechanical loading. In addition, a strategy based on adaptive mesh repartitioning has been introduced into the phase-field method to reduce the computational cost and improve the simulation accuracy by dynamically adjusting the mesh density [19]. This method is particularly effective in dealing with large-scale complex problems, which can significantly reduce the computation time while ensuring the accuracy of crack path prediction. In magnesium alloy corrosion studies, the phase-field method is not only capable of simulating crack initiation and extension, but also capturing corrosion-induced localized stress concentration and material property degradation phenomena [20,21,22,23,24]. Through its unique interface tracking mechanism, the phase-field approach is able to effectively characterize the dynamic evolution process of the metal-environment interface and has successfully achieved the quantitative description of the pitting-cracking transition and crack extension behavior in complex geometries [25,26,27]. It is worth noting that the theoretical framework of this method has been extended to the field of crystalline materials, and scholars have successively established phase-field models that take into account the effects of crystal orientation in single-crystal [28,29] and polycrystalline systems [30,31]. By coupling the phase-field model with a multiphysics field, the electrochemical-mechanical coupling behavior of magnesium alloys in corrosive media can be simulated to reveal the corrosion crack initiation mechanism and its extension law. In addition, the phase-field method is able to simulate the fracture mode transition of magnesium alloys under different environmental conditions, such as the transition behavior from brittle fracture to ductile fracture. These studies provide important theoretical support for life prediction and performance optimization of magnesium alloys in corrosive environments. However, despite the significant progress made by the phase-field method in magnesium alloy corrosion studies, there are still some urgent problems to be solved. For example, there are still limitations for the synergistic damage mechanisms under the coupling of multiphysical fields; the interactions between electrochemical corrosion processes, material microstructural features, and external mechanical loads have not been fully elucidated [28,29,30,31]. How to more accurately characterize the microstructural evolution of magnesium alloys in corrosive environments and how to better integrate the phase-field modeling with experimental data remain the focus of future research [32,33].

To address this challenge, the research in this paper can be carried out in the following three aspects: first, the relationship between the electrode kinetic expression of the secondary current and the residual plastic strain is fitted by the experimentally measured Tafel curves, and the corrosion rate is calculated by using the secondary current field in COMSOL 6.0, a finite element software; second, the model of the phase field is set up in COMSOL and the corrosion results of the secondary current module are fitted to ensure the accuracy of the phase-field model; third, the applicability of the phase-field model in magnesium alloy corrosion research is verified and optimized by combining experiment and simulation. The interface kinetic coefficients in the phase-field model are fitted to ensure the accuracy of the phase-field model. Also, the applicability of the phase-field model in magnesium alloy corrosion research is verified and optimized by combining experiment and simulation.

## 2. Materials and Methods

### 2.1. Technical Route

**Figure 1:**
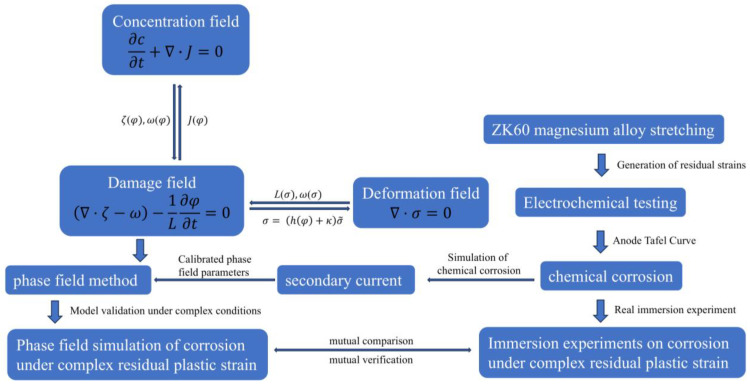
Technical Route.

Figure 1 shows the technology roadmap for this study.

### 2.2. Tensile and Electrochemical Tests

In this section, the relationship between the electrode kinetic expression for the secondary current and the residual plastic strain is fitted by the experimentally measured Tafel curve. The alloy chosen for this thesis is the forged ZK60 magnesium alloy(Qingmei Metals, Dongguan, China), and the specific chemical composition of this alloy is shown in Table 1.

#### 2.2.1. Tensile Test

**Figure 2:**
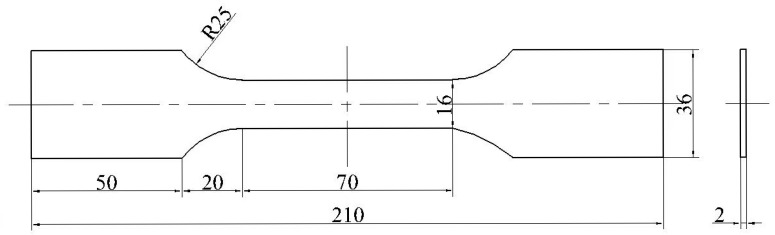
Dimensional Drawing of ZK60 Magnesium Alloy Tensile Specimen.

Referring to the national standard GB/T228.1-2010 [34] to prepare a standard tensile specimen, the shape and size of the specimen are shown in Figure 2.

The loading test machine used in the tensile test setup is the MTS810 material testing system from MTS Systems, Eden Prairie, MN, USA. In order to effectively minimize the stresses and torques caused by poor assembly and other factors, a hydraulic fixture was used to clamp the specimens in the experiments. The strain rate and the corresponding tensile rate were set to 0.0001 s^−1^ and 0.005 mm·s^−1^, respectively, and three parallel specimens were set for each group due to the low strain rate. The specific steps of the test are as follows:

1. Firstly, a matte white primer was coated on the surface of the specimen as the background layer for filming, and then after the primer was cured, a black scattering spot was uniformly sprayed as the marking point for DIC identification;
2. The prepared specimen is mounted to the tensile test platform, which is simultaneously equipped with LED lighting system and high-speed image acquisition device;
3. A high-speed camera is synchronously triggered to shoot when loading is initiated to ensure that the image recording is synchronized with the timing of data acquisition, and the image recording is ended when the test is terminated;
4. An equal interval sampling method was adopted to select samples for analysis, and the deformation field and strain distribution of the specimen were calculated by comparing the spatial displacement of the scattering markers in the time sequence image with the help of Vic-2D7 analysis software.

#### 2.2.2. Electrochemical Test

A cylinder with a base area of 1 cm^2^ and a height of 2.5 mm was cut from the tensile specimen’s gauge section as the electrochemical test sample. On the back of the specimen, a tin soldering process was used to connect the diameter of 0.5 mm pure copper wire, with soldering iron temperature control at 250 ± 10 °C. After soldering, the non-working surface, wire, and solder joint area were coated with an epoxy resin insulation layer and cured at room temperature. The test surface of the specimen needs to be sequentially 1000#, 1200#, 1500#, 2000#, 3000# silicon carbide sandpaper for gradient grinding. During the grinding process, every time the sandpaper label is changed, the specimen is rotated 90° to eliminate the unidirectional scratches generated by the previous pass, and to ensure the uniformity of the grinding. After the final treatment with 3000# sandpaper, the surface was polished with diamond abrasive paste until the surface was smooth and mirrored, and the surface was examined by optical microscope to check the surface morphology, which required that there were no scratches with a length of more than 20 μm or craters with a depth of more than 5 μm in the field of view. After the surface treatment was completed, the specimens were placed in an anhydrous ethanol ultrasonic cleaning tank for 15 min to thoroughly remove the residual abrasive debris and contaminants on the surface, followed by blow-drying with an electric hairdryer, and preserved in a vacuum bag under vacuum to simulate the surface oxidation. The compositions of the Hank’s solution are shown in Table 2.

The electrochemical experiments were carried out using the PARSTAT 4000A electrochemical workstation developed by Ametek, Oak Ridge, TN, USA, with the initial potential set to −1 V (vsOC), the end potential set to 2 V (vsOC), the step set to 1 mV, and the scanning time of 0.01 s per step. A saturated calomel electrode was chosen as the reference electrode for the electrochemical experiments.

### 2.3. Phase-Field Modeling

The chemical property of Mg is quite active. In Hank’s solution, ZK60 magnesium alloy is very easy to undergo hydrogen precipitation corrosion, as shown in Equations (1) and (2). In the reaction process, the anode region of magnesium loses electrons and generates Mg^2+^, and the cathode region obtains the electrons to produce H_2_ and OH^−^, as shown in Equation (3). OH^−^ and Mg^2+^ will be generated by the reaction of Mg (OH)_2_, and the Mg (OH)_2_ can not be dissolved in water, which is the main corrosion products of magnesium alloy. Corrosion products covered on the surface of magnesium alloy can delay the corrosion of the magnesium matrix to some extent.

(1) $$Mg-2e^{-}\to {Mg}^{2+},$$

(2) $$2H_{2}O+2e^{-}\to 2{OH}^{-}+H_{2}↑,$$

(3) $${Mg}^{2+}+2{OH}^{-}\to Mg(OH{)}_{2}↓,$$

In this paper, based on the commercial finite element software COMSOL Multiphysics 6.0 a multiphysics field coupling simulation model of ZK60 magnesium alloy corrosion is established, which comprehensively considers the coupling processes of matter exchange, pha se transformation, and solid mechanics. The dynamic tracking of the corrosion interface is realized by introducing the phase-field variable *φ* to distinguish between the fully corroded phase (*φ* = 0) and the intact solid phase (*φ* = 1); here, the stiffness and mass are completely damaged, and combined with the material transfer field equations. The method avoids the problems of traditional dynamic mesh techniques in dealing with complex results, and at the same time, overcomes the inherent defects of the quadratic current method, such as inefficiency and poor three-dimensional adaptability in calculating complex structures. The application of the phase-field method enables the model to efficiently simulate the natural evolution of the solid-liquid interface during the corrosion process, which significantly improves the calculation accuracy and adaptability of complex geometries [35].

The customized phase-field parameters and material properties are shown in Table 3.

In this paper, the total Helmholtz free energy density can be decomposed into a mechanical $\Phi^{M}$, an electrochemical $\Phi^{ch}$, and an interfacial component *L*. In this paper, the functional form of the free energy of each system is defined, and the compatible intrinsic relationship of each system is derived.

#### 2.3.1. Corrosion Phase Field

The expression for the corrosion phase field in the model is:

(4) $$\left(\nabla \cdot \zeta -\omega \right)-\frac{1}{L}\frac{\partial \varphi }{\partial t}=0,$$

where *L* is the interfacial kinetic coefficient. The negative damage driving force ω and the negative damage flux ***ζ*** are conjugated to the phase field *φ* and the phase-field gradient **∇*φ***. In the phase-field corrosion regime, the negative damage driving force ω is related to the electrochemical free-energy density *Φ*^EC^ and the energy threshold *Φ*^YZ^, and the electrochemical free-energy density *Φ*^EC^ can be approximated by its chemical counterpart *Φ*^ch^:

(5) $$\Phi^{ch}=A{\left[c-h_{1}\left(\varphi \right)\left(c_{se}-c_{Le}\right)-c_{Le}\right]}^{2}+wg\left(\varphi \right),$$

where *w* is the height of the double-well potential function g (*φ*). g (*φ*) is defined as:

(6) $$g\left(\varphi \right)=\varphi^{2}{\left(1-\varphi \right)}^{2},$$

The double-well potential function g (*φ*) satisfies both g (*φ* = 0) = 0 and g (*φ* = 1) = 0. Meanwhile, to satisfy the electrochemical requirements, $h_{1}\left(\varphi \right)$ is defined as:

(7) $$h_{1}\left(\varphi \right)=-2\varphi^{3}+3\varphi^{2},$$

and it is assumed to define a similar curvature A of the free energy density for the solid and liquid phases; c_se_ and c_Le_ can be derived from the equilibrium concentration c_solid_ in the metal and the liquid-phase The saturation concentration c_sat_ in the metal and the saturation concentration c_sat_ in the liquid phase, c_se_ = c_solid_/c_solid_ = 1 is the normalized equilibrium concentration for the solid and c_Le_ = c_sat_/c_solid_ is the normalized equilibrium concentration for the liquid.

The energy threshold *Φ*^YZ^ represents the minimum energy required for corrosion, and can be derived from Equation (8):

(8) $$\Phi^{YZ}=K_{s}\frac{G_{c}}{l_{c}},$$

where *k*_S_, *G*_c_, and *l*_c_ are the energy threshold coefficient, ion transport mobility, and interface characteristic thickness, respectively.

According to Equations (5) and (8), an expression for the negative damage driving force *ω* can be introduced as in (9):

(9) $$\omega =-K_{s}\frac{G_{c}}{l_{c}}-A\left[c-h_{1}\left(\varphi \right)\left(C_{se}-C_{Le}\right)-C_{Le}\right]\left(C_{se}-C_{Le}\right)h_{1}^{\prime }\left(\varphi \right)+wg\prime \left(\varphi \right),$$

while the negative damage flux ***ζ*** is given by Equation (10):

(10) $$\zeta =D_{0}G_{c}l_{c}\nabla \varphi ,$$

Substituting the intrinsic relations (9) and (10) into the phase-field balance, Equation (4) yields the so-called Allen-Cahn equation:

(11) $$\frac{\partial \varphi }{\partial t}+L\left(\omega -{D_{0}G}_{c}l_{c}\nabla^{2}\varphi \right)=0,$$

#### 2.3.2. Matter Transfer Field

The expression for the matter transfer field in the model is:

(12) $$\frac{\partial c}{\partial t}c_{solid}+\nabla \cdot J=0,$$

(13) q=J·n,

where *c* is the dimensionless concentration of Mg ions, ***J*** is the flux defined by Neumann-type boundary conditions, and *q* is the surface flux. According to the Fick’s law type relationship, the flux ***J*** can be expressed as shown in Equation (14) without considering the degradation of the dielectric properties:

(14) $$J=\frac{M}{2A}{c_{solid}}^{2}\nabla \mu =-c_{solid}M\nabla \left[c-h_{1}\left(\varphi \right)\left(c_{se}-c_{Le}\right)-c_{Le}\right],$$

Substituting Equation (12) into the mass transfer equilibrium, Equation (10) yields the equation for the matter transfer field shown in Equation (15):

(15) $$\frac{\partial c}{\partial t}-\nabla \cdot M\left(\nabla \left[c-h_{1}\left(\varphi \right)\left(c_{se}-c_{Le}\right)-c_{Le}\right]\right)=0,$$

#### 2.3.3. Solid Mechanics

The mechanical free energy density *Φ*^M^ of the complete grouping *Φ*^M^ is considered to be the plastic component *Φ*^P^ in this paper, and the plastic component *Φ*^P^ is degraded by the phase field:

(16) $$\Phi^{M}=h_{2}(\varphi )\Phi^{P},$$

Here, the degeneracy function h_2_ (*φ*), which characterizes the transition from the intact solid phase (*φ* = 1) to the destroyed phase (*φ* = 0) with complete stiffness and mass damage, is defined as follows:

(17) $$h_{2}(\varphi )=\varphi^{2},$$

The accumulated plastic strain of the intact solid is computed from the plastic strain tensor $\varepsilon^{p}$ in the intact configuration,

(18) $${\bar{\varepsilon }}^{p}=\int \sqrt{\frac{2}{3}d\varepsilon^{p}:d\varepsilon^{p}},$$

Equation (11) shows that the corrosion dissolution of a material is controlled by the interfacial kinetic coefficient *L*. In this paper, the interfacial kinetic coefficient is considered to be influenced by the cumulative plastic strain ${\bar{\varepsilon }}^{p}$.

Finally, isotropic yielding and associated flow rules are applied. The work hardening of the material is defined by assuming a power-law hardening behavior, using the Swift isotropic hardening model in the small plastic strain model of Equation (19)

(19) $$\tilde{\bar{\sigma }}=\sigma_{y}{\left(1+\frac{E{\bar{\varepsilon }}^{p}}{\sigma_{y}}\right)}^{N},$$

where *E* is the Young’s modulus, $\tilde{\bar{\sigma }}$ is the equivalent stress for the complete configuration, ${\bar{\varepsilon }}^{p}$ is the cumulative plastic strain, $\sigma_{y}$ is the yield stress, and *N* is the strain-hardening index (0 ≤ *N* ≤ 1). The above Swift model parameters were obtained by real experimental fitting.

From (17), the relationship between stress and strain can be as in Equation (20):

(20) $$\sigma =h_{2}\left(\varphi \right)C:\varepsilon^{\mathrm{e}},$$

Taking into account the effect of the phase field and using $\tilde{\sigma }$, the mechanical force balance in Equation (20) can be reformulated as:

(21) $$\nabla \cdot \left[\left(h_{2}\left(\varphi \right)+\kappa \right)\tilde{\sigma }\right]=0,$$

where *κ* is a small positive parameter used to avoid complete degeneracy of the energy and to ensure that the algebraic condition number remains appropriate. *κ* = 1 × 10^−7^ is used throughout the model to ensure convergence.

### 2.4. Corrosion Modeling of Secondary Currents

The anode Tafel curves of magnesium alloy ZK60 with different plastic residual strains were obtained from the polarization curves measured in Section 2.2. The electrochemical corrosion model was established under the secondary current distribution physical field in COMSOL Multiphysics, the anodic equilibrium potential *E*_eqa_ was set as the open circuit potential of the plastic strain-free ZK60, and the local current density $I_{A}$ expression in the electrode dynamics was set as the anodic Tafel equation shown in Equation (22):

(22) $$I_{A}=i_{0}10^{\frac{\eta }{A_{a}}},$$

where *i*_0_ is the anodic exchange current density, *A*_a_ is the anodic Tafel slope, and *η* is the overpotential. *i*_0_ and *A*_a_ can be introduced by the Tafel slope Equation (21):

(23) $$\eta =a+blgI_{A},$$

From Equation (23), $i_{0}=10^{\frac{-a}{b}}$ and $A_{a}=b$, where *a* and *b* are the intercept and slope, respectively, of the fit to the Tafel segment of the kinetic polarization curve.

The electrode reaction at the cathode is Equation (24), the cathodic equilibrium potential *E*_eqc_ is set to the hydrogen precipitation potential of the plastic strain-free ZK60, and the expression for the localized current density $I_{C}$ in the electrode kinetics is set to the cathodic Tafel equation.

(24) $$I_{C}={-i}_{c}10^{\frac{\eta }{A_{c}}},$$

where *i*_c_ is the exchange current density for cathodic hydrogen precipitation and *A*_c_ is the cathodic Tafel slope.

### 2.5. Fitting Phase-Field Dynamics Parameters Based on the Secondary Current Model

In this section, firstly, the corrosion rate of the baseline model is calculated using the secondary current module, and secondly, the model of the same phase field is constructed, and the corrosion kinetic parameter L of the phase field is calibrated by the corrosion results of the secondary current module, which realizes the conversion between electrochemical corrosion kinetics and phase-field theory.

#### 2.5.1. Physical Field Simulations of Secondary Currents with Different Plastic Residual Strains

**Figure 3:**
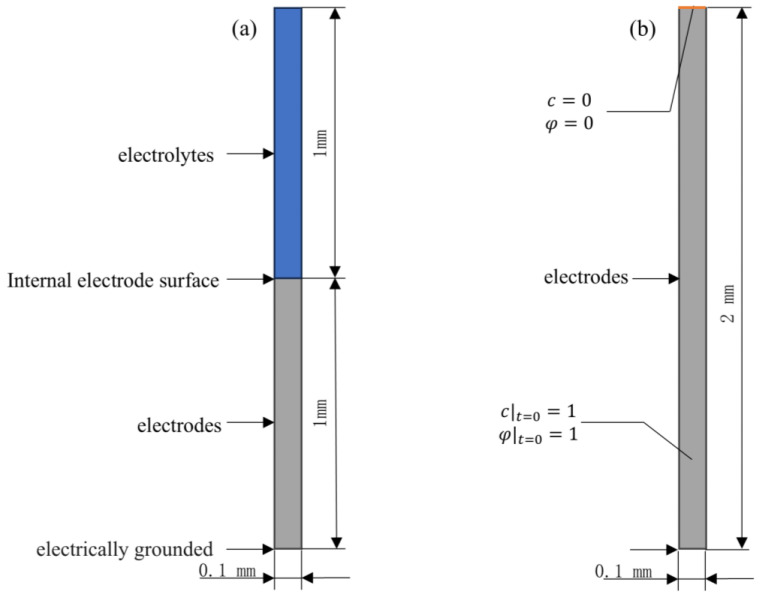
(**a**) Secondary current model, (**b**) Phase-field model.

The electrochemical model is built as shown in Figure 3a, setting up the electrolyte, internal electrode surface, and electrodes in turn, and setting up the anodic and cathodic reaction equations of the internal electrode surface according to the above.

#### 2.5.2. Physical Field Simulations with Different Plastic Residual Strain Phase-Concentration Fields

The phase-field corrosion model (Figure 3b) was built under the general form of a partial differential equation physical field in COMSOL Multiphysics, and the model size was set to 0.1 mm × 2 mm in order to simulate the secondary current module more accurately. The Dirichlet boundary conditions *φ* = 0 and *c* = 0 were applied to the upper surface, and the zero flux conditions were applied to the left, lower, and right sides, and the initial conditions *φ* = 1 and *c* = 1 were applied to the scalar segment. The calibration interface kinetic coefficients *L* are calibrated by comparing the position distributions of the phase-field variable *φ* = 0.5 and the concentration-field variable *c* = 0.5 at the same time step. The calibration model is calibrated so that, in order to satisfy the numerical stability requirements for the coupled solution of the phase-field equation and diffusion equations, the maximum offset of the positions of the phase-field variable *φ* = 0.5 and the concentration-field variable *c* = 0.5 at the same time step is not more than 1% of the size of the computational domain.

#### 2.5.3. Fitting of Secondary Current Field and Phase-Field Results for Different Plastic Residual Strains

Functional relationships between the corrosion interface moving velocity-strain, the interface dynamics coefficient L, and the phase-field interface moving velocity are fitted by Section 2.5.1 and Section 2.5.2, respectively. Since the corrosion interface moving velocity is equivalent to the phase-field interface moving velocity in the phase field, the functional relationship between the strain and the interface dynamics coefficient L can be derived from the above functional relationship, and in order to improve the accuracy of the model, the two sets of relationships are directly inputted into the COMSOL function.

### 2.6. Plastic Residual Strain Corrosion Experiments

#### 2.6.1. Design of Complex Plastic Residual Strain Dogbone Rods

**Figure 4:**
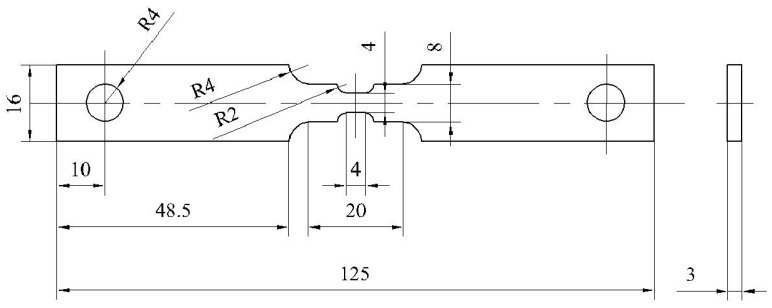
Dimensional Drawing of ZK60 Magnesium Alloy Tensile Specimen with Complex Plastic Residual Strains.

In this study, a progressive cross-section shrinkage structure as shown in Figure 4 is innovatively introduced, and this non-uniform deformation design induces different regions of the specimen to produce differentiated plastic strain fields with good consistency during uniaxial stretching. The procedure for the tensile test of the specimen under DIC is the same as Section 2.2.1.

#### 2.6.2. Immersion Experiments on Complex Plastic Residual Strain Dogbone Rods

**Figure 5:**
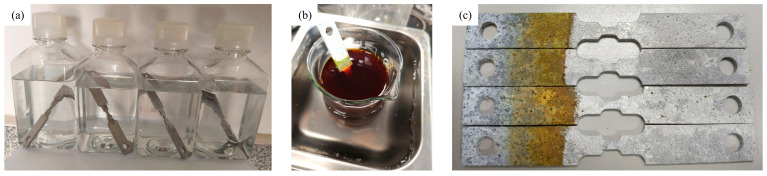
(**a**) Immersion test, (**b**) Chromic acid cleaning, (**c**) Post-cleaning completion.

According to the standard specification of ASTMG31-72 [36], Hank’s balanced salt solution impregnation corrosion experiment was conducted on ZK60 magnesium alloy specimens in this study. The experimental design strictly follows the parameter requirement of a liquid-solid ratio of 1:100, and the constant temperature water bath device equipped with a PID digital temperature control system is used to maintain the solution temperature precisely at 37.3 ± 0.2 °C biomimetic environment, and the experimental period is set to 15 days. As shown in Figure 5a, the experimental setup is schematically shown in order to ensure the stability of the ion concentration of the solution; the fresh corrosive medium is replaced quantitatively every 48 h, and the parameters of the liquid-solid ratio are recalibrated. The container sealing system adopts a semi-open design, through the moderate loosening and closing of the cap to achieve the triple function: effective control of the evaporation rate of the solution, blocking the penetration of environmental pollutants, the timely release of H_2_ gas generated by the corrosion reaction in order to prevent the pressure accumulation of the closed system on the corrosion kinetics of the impact. A pH meter was used to monitor changes in solution acidity and alkalinity at regular intervals during the experiment. After completing the 15-day immersion cycle, the specimens were rinsed with a deionized water jet to remove loose surface adherents. Subsequently, the specimens were treated with an acidic cleaning solution containing 200 g/L of CrO_3_ and 10 g/L of AgNO_3_ at a constant temperature of 60 °C for 10 min (Figure 5b), which was pre-experimentally verified to selectively dissolve magnesium-based corrosion products without affecting the substrate metal; this was followed by a secondary ultrasonic cleaning using ethanol to completely remove the residual chromic acid reagent (Figure 5c).

**Figure 6:**
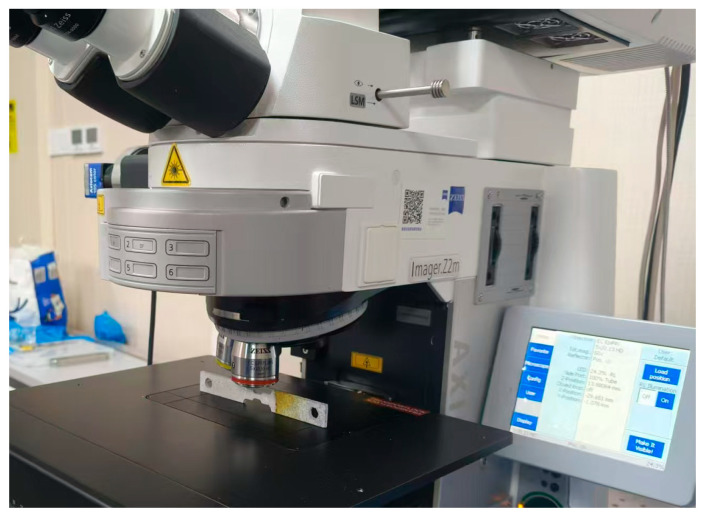
Schematic diagram of ZEISSLSM900 laser confocal inspection.

In order to further quantify the degree of corrosion at different time periods, this study uses a ZEISS LSM900 (Carl Zeiss AG Hesse, Weimar, Germany) laser confocal microscope system to quantitatively characterize the evolution of the surface morphology of magnesium alloy specimens with different values of plastic residual strain during long-term immersion in simulated body fluids. By comparing the three-dimensional surface morphology parameters at different plastic residual strain levels, the intrinsic correlation mechanism reveals the relationship between residual strain and corrosion behavior. Figure 6 shows a schematic of the ZEISSLSM900 laser confocal inspection with the specimen flanked by the shooting surface.

### 2.7. Finite Element Simulation of Complex Plastic Residual Strain Corrosion

#### Two-Dimensional Modeling of Complex Plastic Residual Strain Corrosion

**Figure 7:**
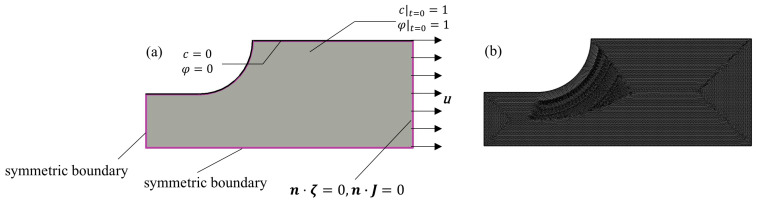
(**a**) 2D Corrosion Model with Complex Plastic Residual Strains, (**b**) Computational Mesh for the 2D Corrosion Model.

According to the complex plastic residual strain tensile specimen of ZK60 magnesium alloy, while considering the economy of finite element calculation, the model only takes the dogbone bar scale distance section in the finite element software COMSOL Multiphysics to establish a two-dimensional model shown in Figure 7a, in order to study the effect of different plastic residual strains on the corrosion rate.

In the previous experiments, immersion experiments were carried out on magnesium alloy specimens with different plastic residual strains, and in order to verify the accuracy of the phase-field model calculations, the model used the same corrosion conditions as the experiments, and the accuracy of the phase field was verified by comparing the results of the experiments with those of the phase-field model calculations.

In Figure 7a, the initial conditions *φ* = 1 and *c* = 1 are applied to the scalar section with the size of 20 mm × 8 mm. The Dirichlet boundary conditions *φ* = 0 and *c* = 0 are applied to the upper surface, the symmetric boundary conditions are applied to the left side and the lower side, and the right side is unloaded after applying the displacement, so as to make the specimen generate the plastic residual strains in the tight section, and at the same time, the zero flux condition is applied to the right side so that the corrosion does not germinate on this side, using the A free triangular mesh as shown in Figure 7b. The maximum size of the mesh is taken to be half of the characteristic thickness lc of the interface in order to ensure the accuracy of the calculation.

## 3. Results

In this study, the effect of different plastic residual strains on the corrosion behavior of ZK60 biomagnesium alloy in physiological solution was investigated through systematic experiments and simulations. The specific work is as follows: five different engineering pre-strains of 0%, 4%, 8%, 12%, and 16% were applied to the ZK60 magnesium alloy specimens using MTS uniaxial tensile machine, the tensile images were captured using a high-speed camera system in the experimental process, and the true stress-true strain curves of the materials were accurately determined by digital imaging technology. The Tafel kinetic polarization curves of ZK60 biomagnesium alloy specimens with different plastic residual strains were measured using an electrochemical workstation. The micro-morphology of the crystals was characterized under an electron microscope. The phase-field corrosion model was established by the finite element software COMSOL Multiphysics: firstly, the measured Tafel kinetic polarization curves were used to fit the anodic electrode reaction parameters required by the secondary current module, and the corrosion rate of the baseline model was calculated by using the secondary current module, and then a model of the same phase field was constructed to calibrate the corrosion kinetics of the phase field by the corrosion results of the secondary current module. Parameter L, the conversion of electrochemical corrosion kinetics to phase-field theory was realized. The accuracy of the phase-field model is verified by quantitatively comparing the roughness of the surface after immersion corrosion of different plastic residual strain specimens and the calculation results of the phase-field model.

### 3.1. Tensile Test Results

**Figure 8:**
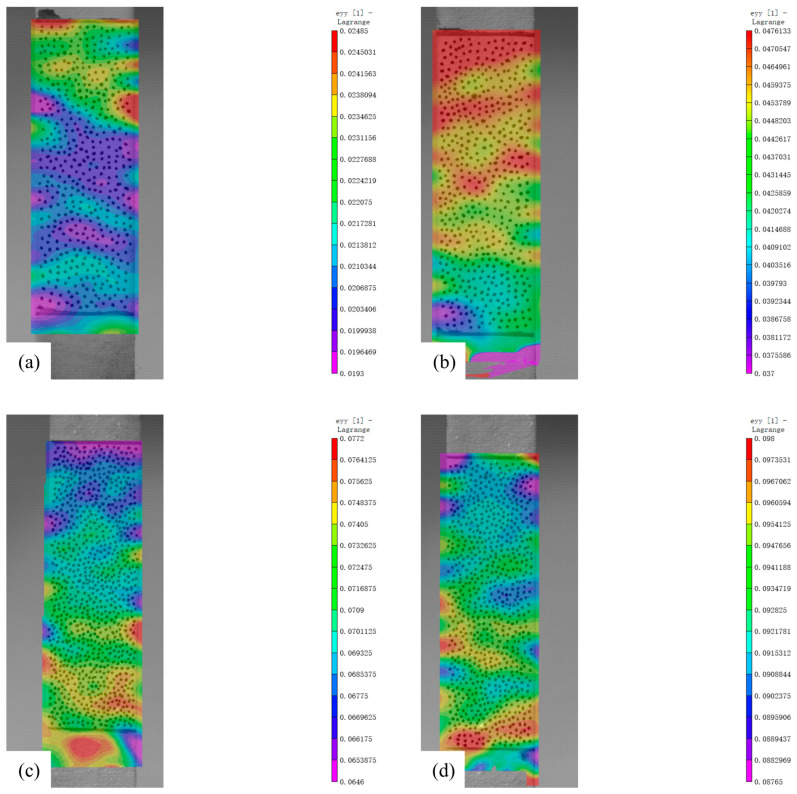
DIC Results Figure: (**a**) Engineering Strain 0.04; (**b**) Engineering Strain 0.08; (**c**) Engineering Strain 0.12; (**d**) Engineering Strain 0.16.

The DIC results of the tensile experiments are shown in Figure 8, and Table 4 shows the average strains measured by DIC.

**Figure 9:**
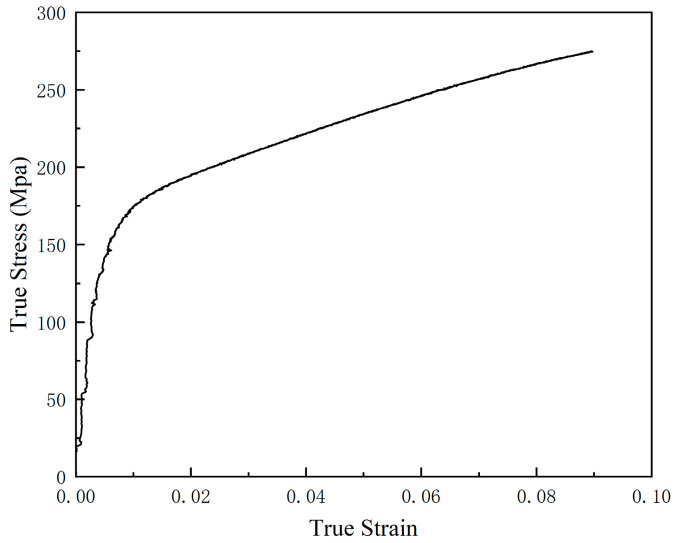
True Stress-True Strain Curve.

In order to get the value of plastic residual strain more accurately, the combination of DIC and MTS is used to calculate σ_true stress_ and ε_true strain_, which are given in the Formulas (23) and (24):

(25) $$\sigma_{true\;stress}=\frac{F}{s}\left(1+\varepsilon_{engineering\;strain}\right),$$

(26) $$\varepsilon_{true\;strain}=\ln\left(1+\varepsilon_{engineering\;strain}\right),$$

where *F* is the value of force collected by the tensile testing machine, *s* is the cross-sectional area of the specimen, and ε_engineering strain_ is the strain value calculated by DIC at the same moment. Figure 9 shows the σ_true stress_-ε_true strain_ plot, the slight fluctuation of the curve in the elastic phase may be due to the error caused by the presence of noise in the DIC sampling data.

**Figure 10:**
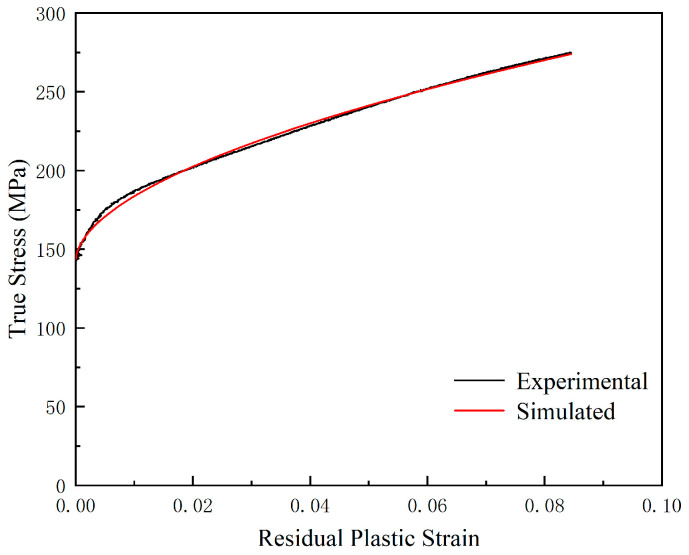
Fitting Results of the Swift Hardening Model.

The Equation (17) Swift model parameters were obtained by curve fitting as described above, the results of which are shown in Figure 10, and the values of the fitted specific parameters are shown in Table 5.

### 3.2. Electrochemical Experiments Results

**Figure 11:**
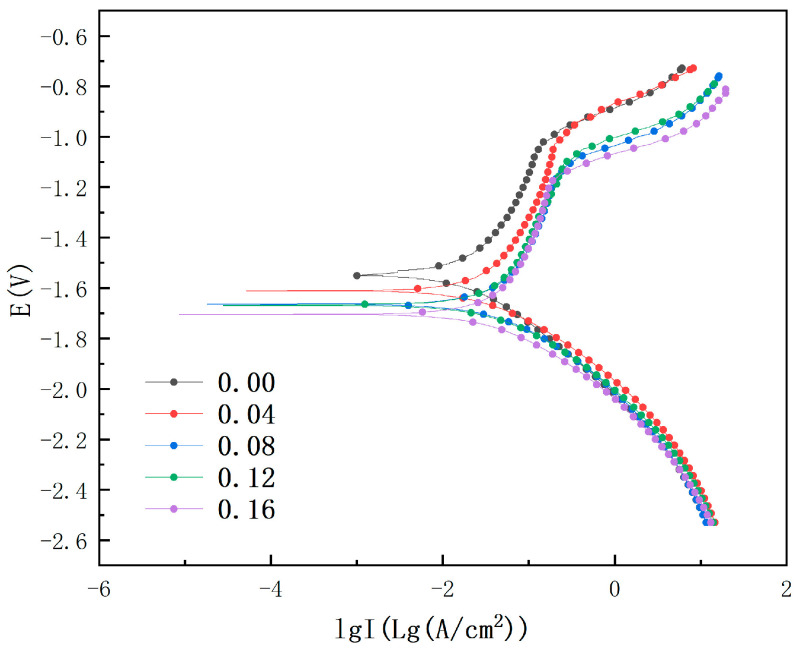
Dynamic polarization curves of ZK60 Magnesium Alloy in Hank’s solution for different engineering strains.

The kinetic potential polarization curves of ZK60 magnesium alloys with different magnitudes of plastic residual strains in Hank’s solution are demonstrated in Figure 11. By studying the electrode reaction process on the working electrode and observing the law of current density change with electrode potential, the polarized corrosion characteristics of the material under different immersion times can be revealed. The polarization curves were fitted using the Tafel extrapolation method, and the fitting results are detailed in Table 6. It can be seen from Figure 11 that the specimens with different plastic residual strains exhibit similar characteristics in the cathodic and anodic polarization curves, which suggests that they have similar electrochemical corrosion mechanisms.

From the polarization curves, it can be observed that the specimens that did not undergo plastic deformation showed the best corrosion resistance with the lowest corrosion current density of 1.841 × 10^3^ A/cm^−2^ and a corrosion potential of −1.551 V. In contrast, the specimens that underwent tensile deformation of 0.02, 0.06, 0.08, and 0.12 increased the corrosion current density to 1.875 × 10^3^ A/cm^−2^, 1.911 × 10^3^ A/cm^−2^, 1.946 × 10^3^ A/cm^−2^, and 1.968 × 10^3^ A/cm^−2^, respectively, while the corrosion potential decreased to −1.611 V, −1.664 V, −1.669 V, and −1.705 V. These data indicate that the corrosion current density of the specimens increases significantly with the increase in the degree of plastic residual strain deformation, and the corrosion potential decreases gradually, indicating a gradual decrease in the corrosion resistance of the material.

The intercept a and slope b required for Equations (20) and (21) are fitted from the kinetic polarization curves in Figure 11, and the fitting results are shown in Table 7.

### 3.3. Results of Fitting the Phase-Field Dynamics Parameters Based on the Quadratic Current Model

In the secondary current physical field, the corrosion rate is reflected by recording the corrosion interface moving position for 4000 s. The corrosion rate of the corrosion interface in the secondary current physical field is shown in Table 8.

**Figure 12:**
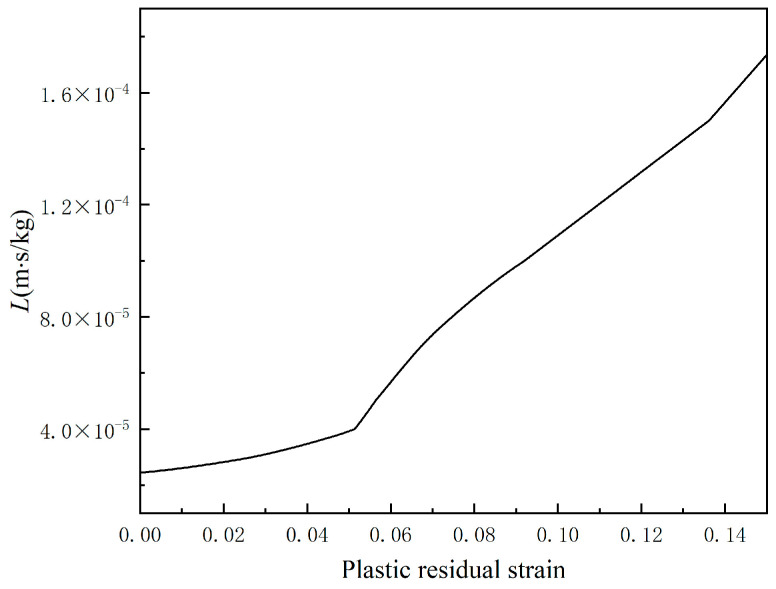
Plastic residual stress-variation interface kinetic coefficient *L* fitting curve.

As shown in Figure 2, the phase field is modeled similarly to the secondary current, and the relationship between the corrosion rate of the corroded interface in the phase field and the interfacial kinetic coefficient L is shown in Table 9. The fitted curves for the kinetic coefficient L of the plastic residual stress-variation interface are shown in Figure 12.

### 3.4. Experimental Results of Plastic Residual Strain Corrosion

#### 3.4.1. DIC Results of Plastic Residual Strain Corrosion Experiments

**Figure 13:**
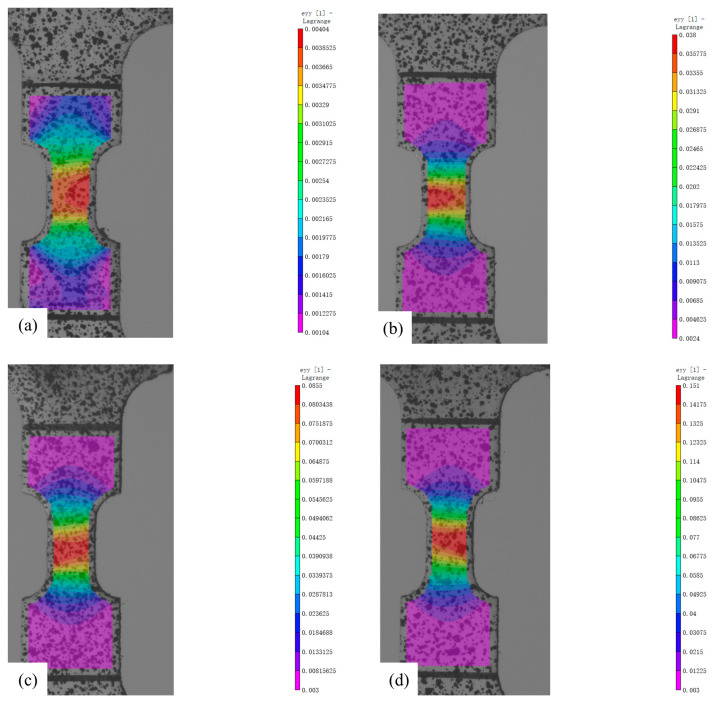
DIC Results of Tensile Testing for ZK60 Magnesium Alloy Specimens with Complex Plastic Residual Strains: (**a**) Engineering Strain 0.02; (**b**) Engineering Strain 0.04; (**c**) Engineering Strain 0.06; (**d**) Engineering Strain 0.08.

The DIC results of the tensile specimens for the experiments with plastic residual strain corrosion are shown in Figure 13, and Table 10 shows the average strains measured by DIC.

#### 3.4.2. Surface Roughness Results of Plastic Residual Strain Corrosion Experiments

**Figure 14:**
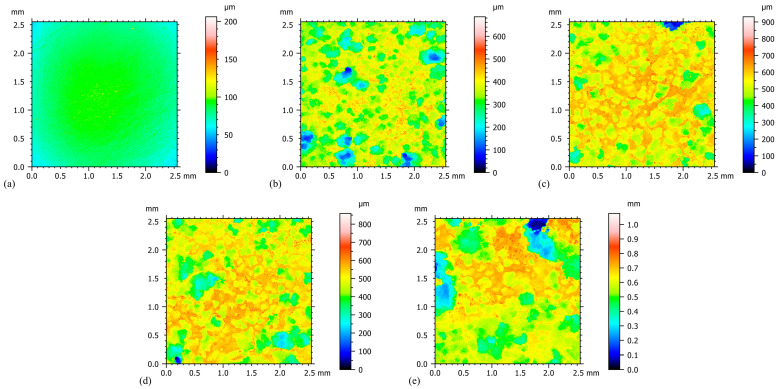
2D Corrosion Morphology of ZK60 Magnesium Alloy with Different Plastic Residual Strains: (**a**) Before Corrosion; (**b**) Engineering Strain 0.02; (**c**) Engineering Strain 0.04; (**d**) Engineering Strain 0.06; (**e**) Engineering Strain 0.08.

The two-dimensional surface roughness evolution shown in Figure 14 indicates that the degree of corrosion on the material surface shows a significant positive correlation with the amount of plastic deformation. From the quantitative data in Table 11, it can be seen that only localized microcorrosion occurs on the surface of the specimen at the stage of small plastic residual strain, with its Sq and Sa maintained at the levels of 67.17 μm and 50.52 μm, respectively. Notably, when the plastic residual strain reaches 0.038, the surface roughening increases, and the Sq and Sa values jump to 79.02 μm and 60.63 μm, respectively, which are 17% and 20% higher than the initial stage. This linear growth trend implies that microstructural destabilization may have occurred in the material after the critical strain value, accelerating the etching process.

**Figure 15:**
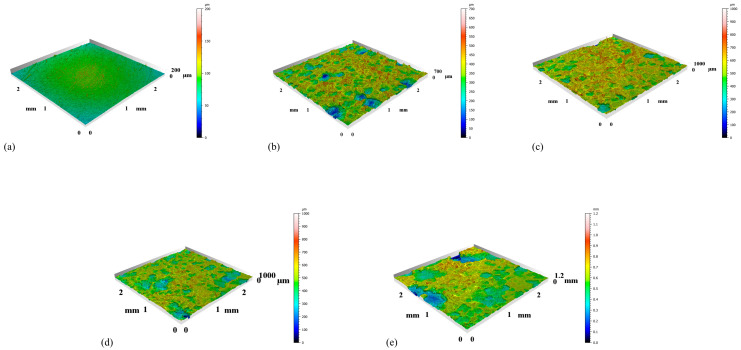
3D Corrosion Morphology of ZK60 Magnesium Alloy with Different Plastic Residual Strains: (**a**) Before Corrosion; (**b**) Engineering Strain 0.02; (**c**) Engineering Strain 0.04; (**d**) Engineering Strain 0.06; (**e**) Engineering Strain 0.08.

The dynamic process of corrosion development can be clearly identified through the analysis of the series of 3D morphology maps (Figure 15). Prior to corrosion, the surface morphology shows a uniform light-green hue, and microcorrosion pits corresponding to roughness values less than 10 μm are randomly distributed due to the polished surface (Figure 15a). As the strain accumulated to 0.04, the surface color spectrum shifted towards the yellow-orange domain, forming contiguous corrosion depressions with a depth of 50–80 μm, but the overall corrosion pattern remained relatively uniform (Figure 15b). When the plastic residual strain reached 0.08, characteristic deep red spots appeared in localized areas, indicating that the corrosion had exceeded 100 μm, suggesting that selective anodic dissolution was occurring in the area of stress concentration (Figure 15d). Eventually, at a plastic residual strain of 0.15, the surface shows a mosaic structure with red-blue tones, and corrosion gullies with a depth of more than 120 μm are interconnected, forming a three-dimensional mesh corrosion channel (Figure 15e).

### 3.5. Plastic Residual Strain Corrosion Simulation Results

#### 3.5.1. Plastic Residual Strain Corrosion Simulation Plastic Residual Strain Results

**Figure 16:**
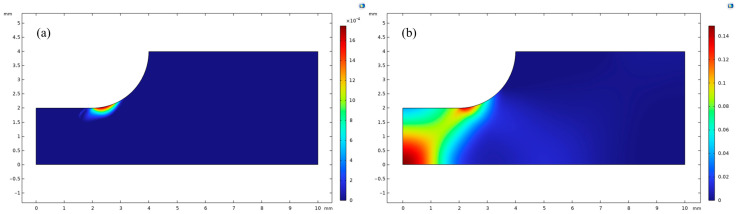
(**a**) 80 s Plastic Residual Strain; (**b**) 1000 s Plastic Residual Strain.

The plastic residual strain distribution of the specimen is shown in Figure 16; Figure 16a for 80 s plastic residual strain, and Figure 16b for 1000 s plastic residual strain.

Figure 16 can be observed that when the specimen is subjected to axial tensile stretching, the stress distribution in the scalar section exhibits obvious nonuniformity. The necking down design of the specimen scale section makes the stress highly concentrated in the geometrical transition zone, forming a large stress gradient, which generates different degrees of plastic strains.

From the distribution of plastic residual strain, it can be seen that in the region of the parallel section away from the clamping end, the plastic residual strain tends to be uniformly distributed; however, due to the sudden change of the cross-section in the necking part, a complex three-dimensional stress field will be generated, and the maximum principal stresses tend to appear in the junction of the necking arc and the straight section, which will lead to the phenomenon of concentration of the plastic residual strain in this area (Figure 16a). As the material enters the plastic deformation stage, the isotropic property prompts the stress redistribution, resulting in the simultaneous existence of circumferential tensile stress and radial compressive stress in the necking region. From the analysis of the finite element simulation results, it can be found that the stress contours in the elastic stage are symmetrical spindle-shaped, while after yielding, due to the effect of plastic deformation, the necking region will form a high stress concentration zone similar to the butterfly shape (Figure 16b), which can also be explained by St. Venant’s principle.

In Figure 16, the maximum plastic residual strain in the necking section reaches 0.14, and the maximum plastic residual strain in the un-necked section is almost 0. There is a good agreement between the simulation result of the plastic residual strain and that of the immersion experiment by DIC analysis, and it appears that the simulation model accurately controls the immersion experiment.

#### 3.5.2. Plastic Residual Strain Corrosion Simulation Corrosion Results

**Figure 17:**
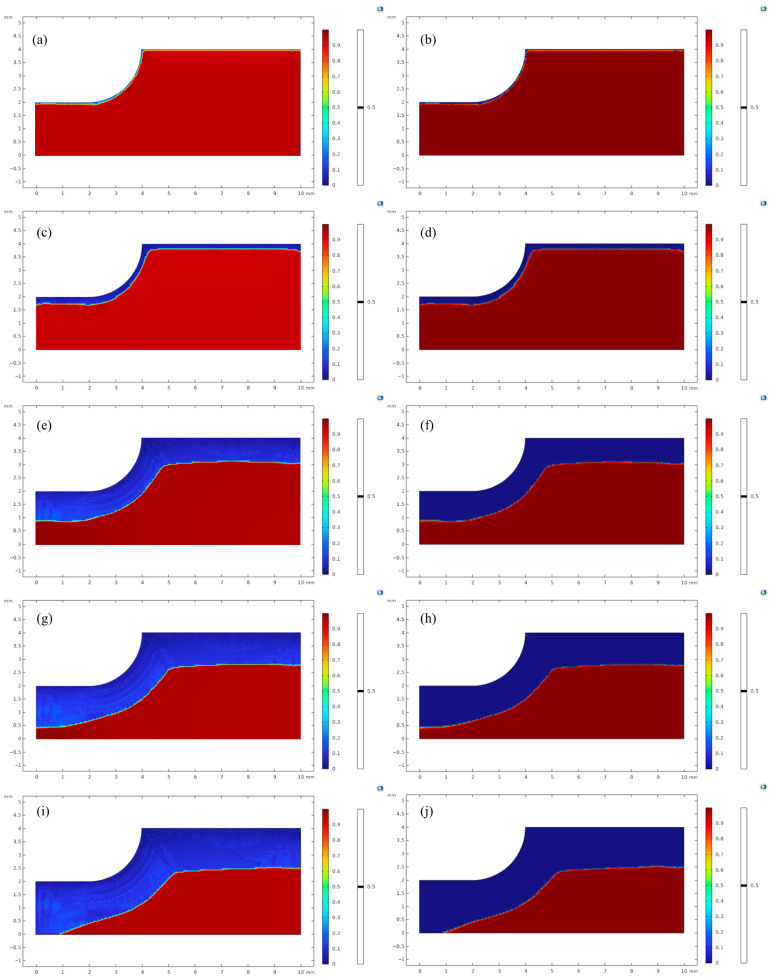
Corrosion Results Figure: (**a**) t = 1.3006 × 10^6^ s, *c*; (**b**) t = 1.3006 × 10^6^ s, *φ*; (**c**) t = 1.0002 × 10^7^ s, *c*; (**d**) t = 1.0002 × 10^7^ s, *φ*; (**e**) t = 1 × 10^8^ s, *c*; (**f**) t = 1 × 10^8^ s, *φ*; (**g**) t = 1.6 × 10^8^ s, *c*; (**h**) t = 1.6 × 10^8^ s, *φ*; (**i**) t = 2.3456 × 10^8^ s, *c*; (**j**) t = 2.3456 × 10^8^ s, *φ*.

The results of the effect of complex plastic residual strain on the corrosion behavior are shown in Figure 17.

In the early stage of corrosion, due to the significant stress concentration and plastic residual strain in the necking region, the corrosion rate in this part is significantly higher than in other regions, showing a more violent corrosion reaction. Its corrosion depth reaches a maximum of 120 μm, and at the same moment, the average corrosion depth of the necking section is about 100 μm, while that of the non-necking section is only 60 μm. The corrosion results of this simulation are in good agreement with the surface roughness results of the immersion corrosion experiments. With the increase of corrosion time to t = 1.0002 × 10^7^ s, the surface corrosion thickness has reached about 0.4 mm; with the increase of corrosion time to t = 1 × 10^8^ s, the corroded surface position of the necking area has already entered the high residual strain area (Figure 17b), and the surface has been corroded by 1.2 mm from the finite element results, and when the corrosion time increases to t = 1.6 × 10^8^ s (Figure 17h), the corrosion depth of the non-necking section is about 100 μm, while the non-necking section is only 60 μm (Figure 17h), the surface has been corroded by 1.6 mm, and the component has entered the critical failure state. When the corrosion time increases to 2.3456 × 10^8^ s (Figure 17j), the necking area has been completely corroded, the member has already failed, and the structure loses its load-bearing capacity.

In the simulation of the corrosion evolution process, comparing the interface positions of the phase-field variable *φ* = 0.5 and the concentration field variable *c* = 0.5, the results show that they exhibit good agreement at the same time step. Quantitative analysis shows that the deviation of the two positions does not exceed 1% of the length of the calculated region. This result not only verifies the self-consistency of the model in describing the kinetic behavior of the corrosion front but also shows that the coupled solution of the phase-field equation and the diffusion equation has high numerical stability.

## 4. Discussion

### 4.1. Discussion of Corrosion of Stress-Concentrated Components

Previous studies have analyzed the corrosion behavior of ZK60 magnesium alloy under complex plastic residual strain in tension. However, in practical medical applications, bio-magnesium alloy components often suffer from localized stress concentrations due to geometrical complexity, multidirectional stress distribution, and processing defects, leading to more significant plastic residual strains. Such strains may accelerate material failure and cause irreversible damage to human tissues.

**Figure 18:**
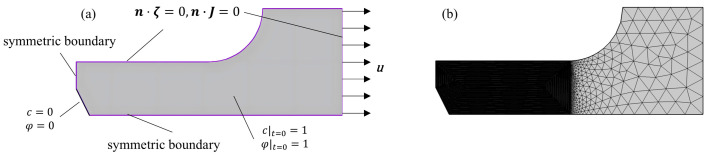
(**a**) 2D model for residual strain corrosion at strain concentration zones, (**b**) Finite element mesh for the 2D residual strain corrosion model at strain localization zones.

Therefore, in order to accurately simulate the corrosion characteristics under such conditions and to predict the service time of magnesium alloys with strain concentrations, a specimen with a beveled notch structure was designed in this study, which is effective in creating a high value of plastic residual strain in the notched region during stress. Based on the design of the specimen, a two-dimensional corrosion simulation model shown in Figure 18 was established in the finite element software COMSOL Multiphysics, and in order to simplify the calculation, the phase-field model was established by taking only the scalar segment, and a symmetric boundary was used to reduce the amount of calculation.

During the modeling process, the initial parameters *φ* = 1 and *c* = 1 were set for the 20 mm × 8 mm spacing section, and the boundary conditions were set as follows: Dirichlet boundary conditions *φ* = 0 and *c* = 0 were used for the sloped area, zero flux constraints were implemented on the upper surface, symmetric conditions were applied to the left and lower boundaries, and displacement constraints and zero flux conditions were applied to the right boundary at the same time. The meshing is done with free triangular cells (Figure 18b), and the maximum size of the mesh is controlled to be 50% of the interface feature thickness lc in order to ensure the computational accuracy.

**Figure 19:**
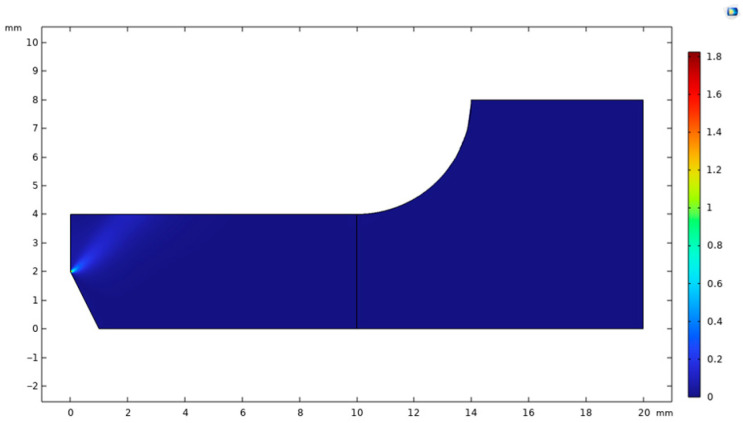
1000 s Plastic Residual Strain.

Figure 19 shows the characteristics of the plastic residual strain distribution of the specimen under axial tensile action. During the stretching process, the stresses in the scalar section show obvious gradient changes, especially in the notched part, with abrupt geometry changes exhibiting a strong stress concentration phenomenon. The stress level in this region is significantly higher than that in other parts, and rises sharply with the approach to the notch tip, forming a typical stress singularity region. This stress concentration effect leads to significant plastic deformation in the notch tip region, forming a localized high-strain zone. Remarkably, the plastic flow properties of the material allow the actual stress levels in this region to be redistributed, thus avoiding the theoretically predicted infinite stress state. The results of the residual strain distribution show that when a displacement load is applied at the right boundary, a significant strain concentration occurs in the defect tip region, with a maximum residual strain value of 1.8. Despite the small distribution of the high-strain region, the specimen as a whole maintains its structural integrity. However, plastic deformation resulted in microstructural changes in the tip region, including increased dislocation density, elevated grain boundary energy, and redistribution of the internal stress field. Together, these changes enhance the electrochemical activity of the material, thereby accelerating the corrosion process. As a result, in the early stage of corrosion, rapid corrosion occurs preferentially in the tip region and gradually expands to the neighboring regions with higher residual strains to form large voids.

**Figure 20:**
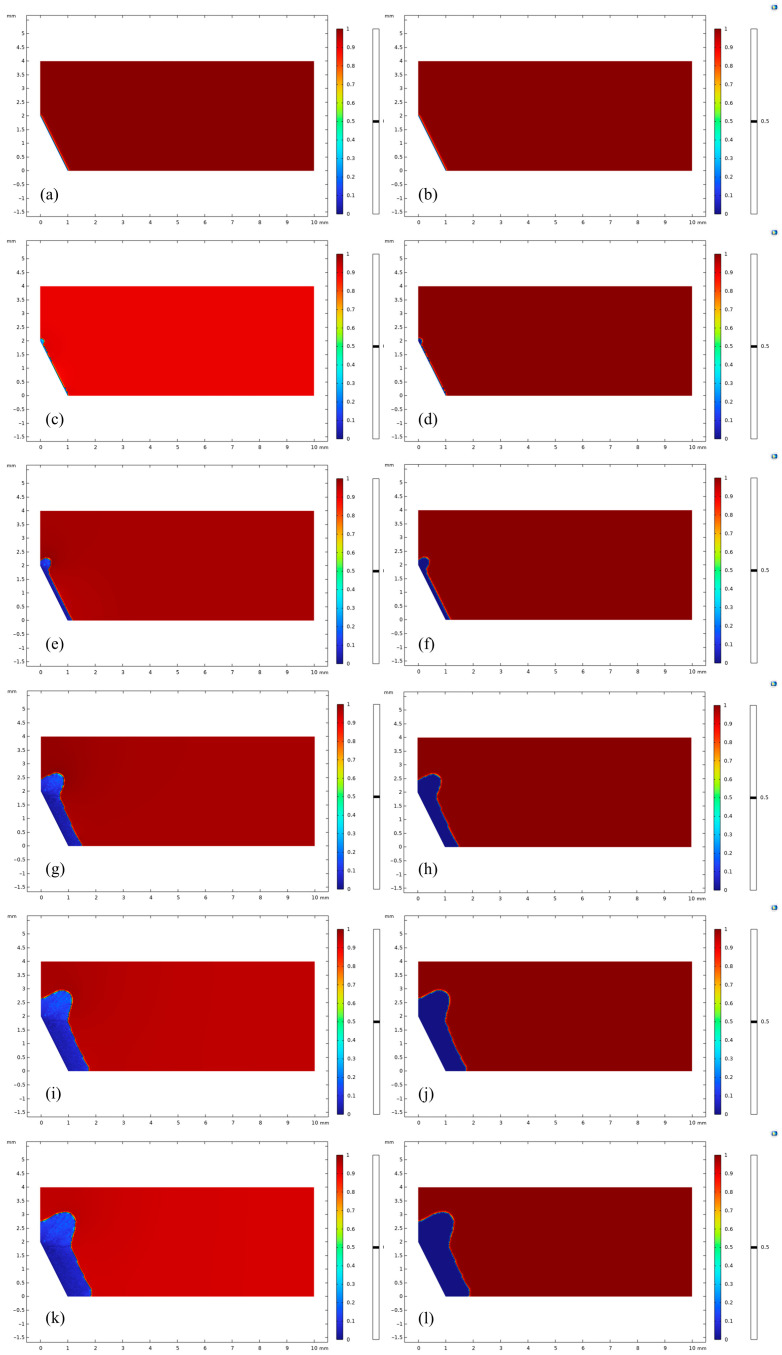
Corrosion Results with Residual Plastic Strain: (**a**) *t* = 1 × 10^3^ s, *c*; (**b**) *t* = 1 × 10^3^ s, *φ*; (**c**) *t* = 1.297 × 10^6^ s, *c*; (**d**) *t* = 1.297 × 10^6^ s, *φ*; (**e**) *t* = 1.0009 × 10^7^ s, *c*; (**f**) t = 1.0009 × 10^7^ s, *φ*; (**g**) *t* = 5.0005 × 10^7^ s, *c*; (**h**) *t* = 5.0005 × 10^7^ s, *φ*; (**i**) *t* = 1.0001 × 10^8^ s, *c*; (**j**) t = 1.0001 × 10^8^ s, *φ*; (**k**) *t* = 1.3322 × 10^8^ s, *c*; (**l**) *t* = 1.3322 × 10^8^ s, *φ*.

Figure 20 demonstrates the significant effect of plastic residual strain in strain-concentrated members on the corrosion characteristics of the material. The phase and concentration fields before corrosion are shown in Figure 20c,d, with the displacement applied at 1 × 10^3^ s and the interface position at 0 mm. During the initial stage of corrosion (Figure 20c,d), the strain-concentrated region shows more active corrosion, with a maximum corrosion depth of 20 μm. Comparison of corrosion in the region where the plastic residual strain is not generated reveals that there is almost no corrosion, which is consistent with the plastic residual strain. As the corrosion continues, when the time reaches 1.0009 × 10^7^ s, the corrosion layer of about 0.2 mm is formed on the surface of the material without plastic residual strain, and the residual strain concentration area already appeared void, which increases the contact area of magnesium alloy and corrosive liquid during the corrosion process, further aggravating the corrosion; finally, at 1.3322 × 10^8^ s, the corrosion thickness of the surface of the material without plastic residual strain reaches 1 mm, and the corrosion thickness of the surface of the material with plastic residual strain reaches 1 mm, which is consistent with the results of plastic residual strain. Material surface corrosion thickness reaches 1 mm, and the tip has produced a huge void; the member will be corroded due to the loss of structural load-bearing capacity.

In the corrosion evolution simulation, by comparing the position distribution of the phase-field variable *φ* = 0.5 and the concentration field variable *c* = 0.5 at the same time step, it is found that the interface positions of the two are highly coincident with each other, and the maximum offset is not more than 1% of the computational domain size. Further analysis shows that the error mainly comes from the numerical dissipation introduced by the grid discretization rather than the deviation of the physical assumptions of the model itself, thus confirming the reliability of the established model.

**Figure 21:**
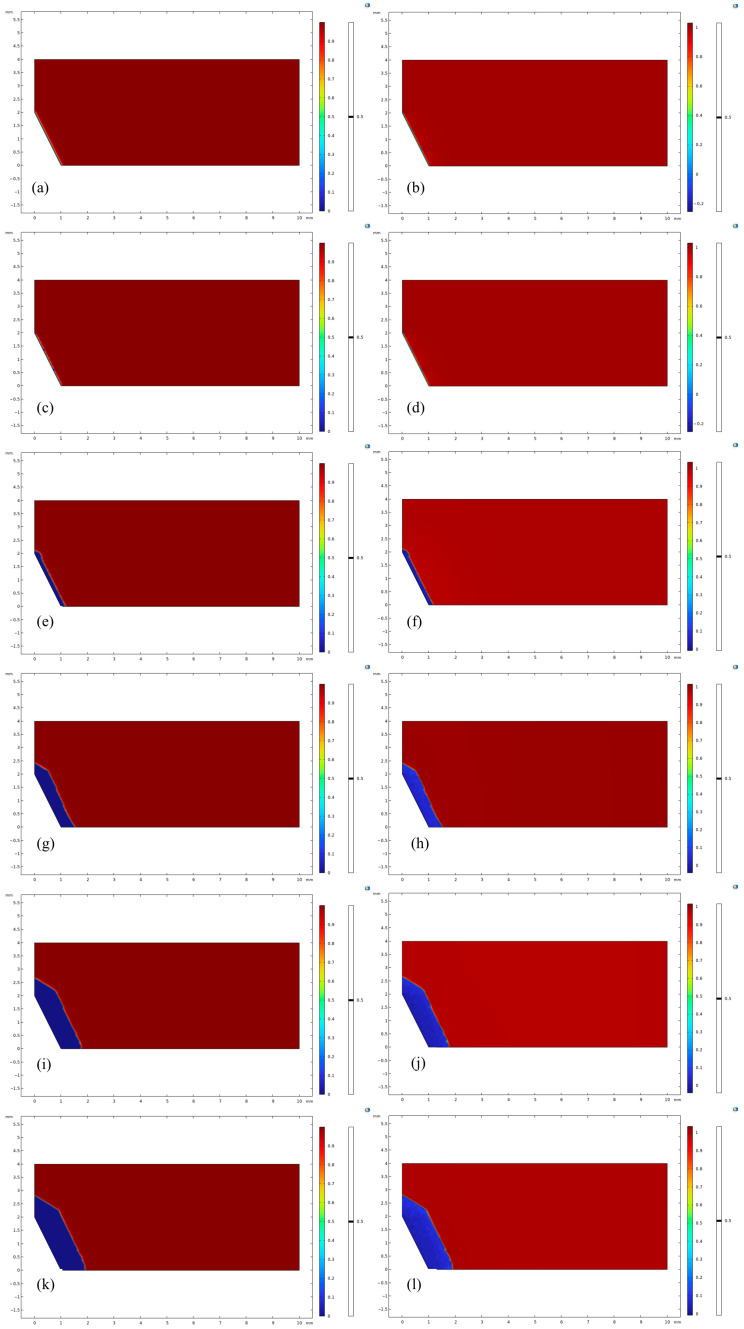
Corrosion Results without Residual Plastic Strain: (**a**) t = 1 × 10^3^ s, *c*; (**b**) t = 1 × 10^3^ s, *φ*; (**c**) t = 1.297 × 10^6^ s, *c*; (**d**) t = 1.297 × 10^6^ s, *φ*; (**e**) t = 1.0009 × 10^7^ s, *c*; (**f**) t = 1.0009 × 10^7^ s, *φ*; (**g**) t = 5.0005 × 10^7^ s, *c*; (**h**) t = 5.0005 × 10^7^ s, *φ*; (**i**) t = 1.0001 × 10^8^ s, *c*; (**j**) t = 1.0001 × 10^8^ s, *φ*; (**k**) t = 1.3322 × 10^8^ s, *c*; (**l**) t = 1.3322 × 10^8^ s, *φ*.

Figure 20 and Figure 21 compare the corrosion characteristics of the members under the influence of plastic-free residual strain and plastic residual strain at the same dimensions. The material tip shows uniform corrosion in the absence of plastic residual strain, which is significantly lower than in the presence of plastic residual strain. A comparison of Figure 20 and Figure 21 shows that plastic residual strains generated during machining result in faster corrosion at the tip of the structure.

### 4.2. Corrosion SEM Result Plots with Residual Plastic Strain Distribution Discussion

**Figure 22:**
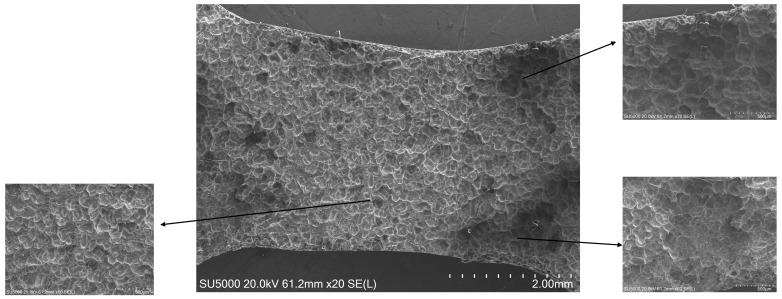
SEM Morphology of ZK60 Magnesium Alloy with Different Plastic Residual Strains.

The microscopic morphology of the corroded surface of ZK60 magnesium alloy immersed in Hank’s solution for 15 days was observed using scanning electron microscopy. As shown in Figure 22, a large number of white second-phase distributions were visible on the specimen surface, while long corrosion grooves of different depths and shades were present on the substrate. The mechanism of this phenomenon is as follows: when the magnesium alloy is in contact with Hank’s solution, the potential difference between the second phase and the substrate will form a corrosion electric couple pair, leading to the dissolution of the magnesium substrate as the anode. At the same time, hydrogen generated from the cathodic reaction escapes from the surface and causes a localized pH increase of the solution, prompting the generation of Mg(OH)_2_ precipitates on the sample surface. However, due to the precipitating effect of hydrogen and the presence of Cl^−^ in the solution, these precipitates are difficult to attach stably, and eventually, cracking or spalling occurs. The cracks formed provide a channel for the diffusion of corrosive media and ions, causing the magnesium substrate to be continuously exposed to the corrosive environment, which results in an inhomogeneous corrosion morphology on the surface. With the prolongation of the immersion time, the continuous dissolution of the magnesium matrix will lead to the original dispersion of the second phase due to the loss of support and the occurrence of fragmentation or detachment, and the formation of corrosion holes of varying sizes. The further expansion and interconnection of these holes will eventually develop into larger corrosion pits. By comparing the distribution of plastic residual strain in Figure 16 with the SEM image after 15 days of immersion corrosion, it can be found that the corrosion pits in the necking region with larger plastic residual strain have interconnected with each other to form larger sized corrosion pits, and their corrosion degree is significantly higher than that in the region with smaller residual strain.

## 5. Conclusions

In this study, a numerical analysis model capable of accurately simulating the service behavior of ZK60 biodegradable magnesium alloy in human physiological solution is constructed based on the coupled multiphysics field modeling method and the kinetic theory of phase-field corrosion damage. The model focuses on the mechanism of plastic residual strain accumulation on the corrosion inhomogeneity of the material and its resulting early failure, and establishes a new multiphysics field coupled simulation framework by integrating the electrochemical corrosion theory with the phase-field approach. The specific work of this paper is summarized as follows:

1. Uniaxial tensile pre-deformation was applied to ZK60 magnesium alloy with 0–16% engineering pre-strain. The true stress-strain curves were accurately determined using high-speed camera systems and digital image correlation techniques, establishing a benchmark for calibrating interface kinetic parameters in phase-field models. Electrochemical tests and microstructural characterization revealed that plastic residual strain enhances local electrochemical activity, elucidating the quantitative correlation mechanism between strain and corrosion rate.
2. A phase-field corrosion model was developed based on COMSOL’s electrochemistry module, where secondary current distribution was employed to calibrate corrosion kinetic parameters, bridging electrochemical theory with phase-field methodology. Innovatively replacing traditional secondary current methods with phase-field approaches effectively addressed challenges, including poor adaptability to complex 3D structures and low computational efficiency. The calibrated model demonstrates rapid adaptability to different magnesium alloy systems, significantly enhancing engineering applicability.
3. Model reliability was verified through 15-day immersion experiments with uniaxial tensile specimens, showing good agreement between surface roughness evolution and simulation results. This model effectively characterizes the regulatory effect of plastic residual strain on biodegradable medical materials degradation behavior, providing a robust numerical analysis tool for service life prediction and structural optimization of medical devices such as degradable vascular stents.

## Figures and Tables

**Table 1:** Chemical composition of ZK60 magnesium alloy.

Element	Mg	Zn	Cr	Mn
wt.%	Balance	5.5~6.5	0.3~0.8	0.3~0.8

**Table 2:** Composition and proportion of Hank’s solution (mol/L).

**NaCl**	**KCl**	**CaCl_2_**	**Na_2_HPO_4_·7H_2_O**
0.137	0.00537	0.00126	0.00034
**NaHCO_3_**	**KH_2_PO_4_**	**C_6_H_12_O_6_**	**MgSO_4_·7H_2_O**
0.00417	0.00044	0.00555	0.00081

**Table 3:** Physical Parameters Table.

Parameters	Value	Unit
Young’s modulus *E*	40,000	MPa
Poisson’s ratio *μ*	0.35	1
Interfacial characteristic thickness *l*_c_	0.1	mm
Free energy density curvature *A*	53.5	N/mm^2^
Height of the double well potential *w*	33.3	N/mm^2^
Average concentration of metal *c*_solid_	72,500	mol/m^3^
Average saturation concentration *c*_sat_	2600	mol/m^3^
Energy threshold coefficient *k*_s_	0.2	1
Surface energy density *G*_c_	0.0002992	mJ/mm^2^

**Table 4:** DIC Measurement Results.

Tensile Displacement (mm)	Strain (1)
0.2	0.022342
0.4	0.047290
0.6	0.070757
0.8	0.093166

**Table 5:** Fitting Parameters Table of the Swift Hardening Model.

Swift Model Parameters	Fitted Value
$\sigma_{y}$	120 (MPa)
*N*	0.152

**Table 6:** Fitting Results of Potentiodynamic Polarization Curve Parameters.

Engineering Strain (1)	*I*_corr_ (A/cm^2^)	*E*_corr_ (V)
0.00	1.841 × 10^3^	−1.551
0.04	1.875 × 10^3^	−1.611
0.08	1.911 × 10^3^	−1.664
0.12	1.946 × 10^3^	−1.669
0.16	1.968 × 10^3^	−1.705

**Table 7:** Fitted Parameters from Potentiodynamic Polarization Curves.

Tensile Displacement (mm)	a (V)	b (V)
0.0	1.06604	0.29408
0.2	1.05269	0.28934
0.4	1.03017	0.28135
0.6	1.00718	0.27320
0.8	0.98400	0.26498

**Table 8:** Corrosion Results from Secondary Current Distribution Module.

Plastic Residual Strain (1)	15-Day Corrosion Interface Moving Position (mm)
0.000000	0.0639
0.022342	0.0687
0.047290	0.0779
0.070757	0.0919
0.093166	0.1014

**Table 9:** Phase-Field Corrosion Simulation Results.

15-Day Phase-field Interface Shift Position (mm)	*L* (m·s/kg)
0.038	0.00001
0.059	0.00002
0.070	0.00003
0.080	0.00004
0.083	0.00005
0.101	0.00010
0.116	0.00015
0.126	0.00020

**Table 10:** DIC Results of Tensile Testing for ZK60 Magnesium Alloy Specimens with Complex Plastic Residual Strains.

Tensile Displacement (mm)	Strain (1)
0.4	0.004
0.8	0.038
1.2	0.085
1.6	0.150

**Table 11:** Surface Roughness after Immersion Corrosion.

Strain (1)	*S*_q_ (μm)	*S*_a_ (μm)
0.004	67.17	50.52
0.038	79.02	60.62
0.080	92.49	69.41
0.150	123.16	97.29

## Data Availability

The main data supporting the findings of this study are available within the article. Extra data are available from the corresponding author upon reasonable request.
